# Supplementary material for: Nomogram for Predicting Survival in Patients Treated with Liposomal Irinotecan Plus Fluorouracil and Leucovorin in Metastatic Pancreatic Cancer
Source: Cancers (Basel). 2019 Jul 28;11(8):1068. doi: 10.3390/cancers11081068 (PMC6721419; doi:10.3390/cancers11081068)
Supplement: Supplementary file 1 [file cancers-11-01068-s001.pdf]

Supplementary Materials

Nomogram for Predicting Survival in Patients Treated with Liposomal Irinotecan Plus Fluorouracil and Leucovorin in Metastatic Pancreatic Cancer

Li-Tzong Chen, Teresa Macarulla, Jean-Frédéric Blanc, Beloo Mirakhur, Floris A. de Jong, Bruce Belanger, Tanios Bekaii-Saab and Jens T. Siveke

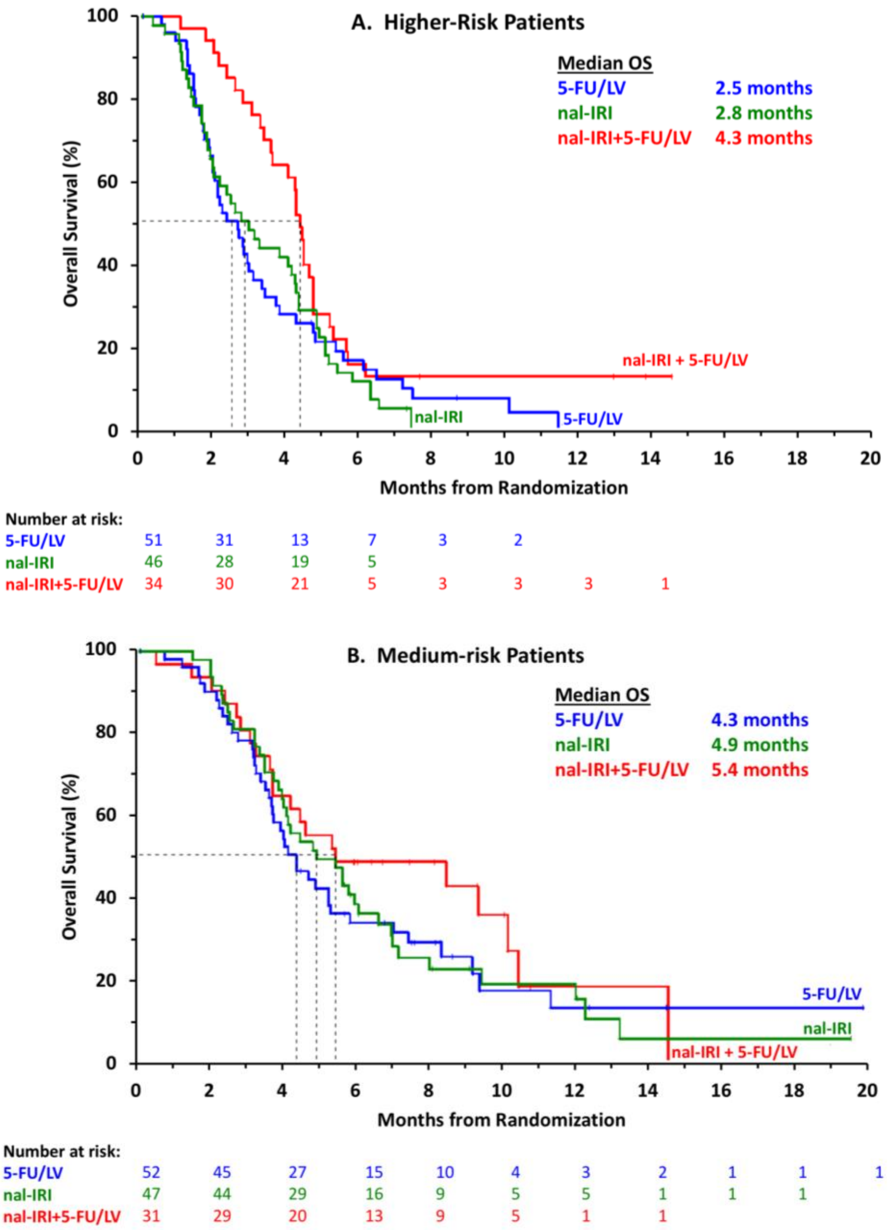

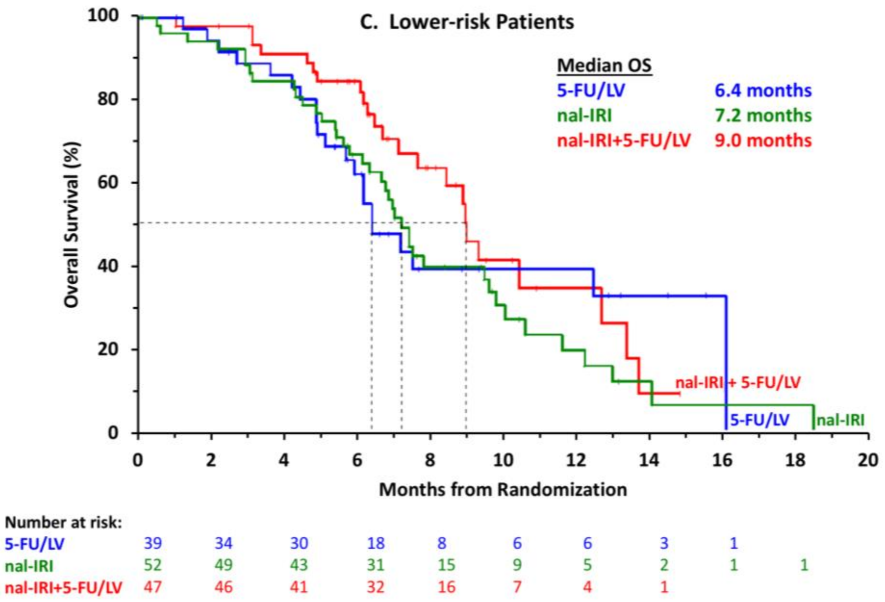

Figure S1. Kaplan–Meier survival curves showing overall survival probability stratified by treatment arm for (**A**) higher-risk patients, (**B**) medium-risk patients, and (**C**) lower-risk patients. 5-FU/LV, 5-fluorouracil/leucovorin; nal-IRI, liposomal irinotecan.

© 2019 by the authors. Licensee MDPI, Basel, Switzerland. This article is an open access article distributed under the terms and conditions of the Creative Commons Attribution (CC BY) license (http://creativecommons.org/licenses/by/4.0/).
